# Physical activity level measured by accelerometry and physical fitness of schoolchildren

**DOI:** 10.1590/1984-0462/2023/41/2021230

**Published:** 2022-09-09

**Authors:** José Matheus Oliveira Estivaleti, Raiany Rosa Bergamo, Luís Carlos de Oliveira, Diana Carolina Gonzalez Beltran, João Pedro da Silva, Maurício dos Santos, Victor Keihan Rodrigues Matsudo

**Affiliations:** a Center for Studies of the Physical Fitness Research Laboratory of São Caetano do Sul, São Caetano do Sul, SP, Brazil.

**Keywords:** Physical activity, Accelerometry, Physical fitness, Students, Atividade física, Acelerometria, Aptidão física, Crianças

## Abstract

**Objective:**

To describe and compare physical fitness variables according to compliance with the recommendations of physical activity, measured by accelerometry.

**Methods:**

The sample gathered 120 students, 57 boys and 63 girls aged 9 to 11 years. The variables analyzed were: weight, stature, BMI, skinfolds, waist circumference, agility, flexibility, speed and strength of upper and lower limbs, and abdominal strength. Physical activity was measured objectively using an accelerometer. The students were divided into two groups: “complies with recommendations” (≥60min/day) and “does not comply with recommendations” (<60min/day). To verify the normality of the data, the *Kolmogorov-Smirnov* test was used. The mean values of students who do or do not comply with the physical activity recommendation were compared using Student's *t* and U-Mann Whitney tests. The level of significance was set at p<0.05.

**Results:**

The students who followed the recommendation showed significantly lower values compared to those who did not for adiposity (sum of 7 skinfolds); body weight; body mass index (BMI) and abdominal strength. No significant differences were found in the variables of speed and agility, and the upper limbs’ strength was greater in subjects who did not comply with recommendations.

**Conclusions:**

Students who complied with physical activity recommendations had better body composition and more abdominal strength than those who did not.

## RESUMO

**Objetivo:**

Descrever e comparar as variáveis da aptidão física de acordo com o cumprimento da recomendação de atividade física, medida por acelerometria.

**Métodos:**

A amostra foi composta de 120 escolares, sendo 57 meninos e 63 meninas, de 9 a 11 anos de idade. As variáveis analisadas foram: peso, estatura, índice de massa corpórea (IMC), dobras cutâneas, circunferências, agilidade, flexibilidade, velocidade e força de membros superiores, inferiores e abdominal. A atividade física foi mensurada de forma objetiva por meio de acelerômetro. Os escolares foram divididos em dois grupos: “cumpre a recomendação” (≥60min/dia) e “não cumpre a recomendação” (<60min/dia). Para verificar a normalidade dos dados, foi utilizado o teste de Kolmogorov-Smirnov. A comparação dos valores médios dos escolares que cumprem ou não a recomendação de atividade física foi feita pelos testes *t* de Student e U de Mann-Whitney. O nível de significância adotado foi de p<0,05.

**Resultados:**

Os escolares que cumpriram a recomendação, comparados com os que não cumpriram, apresentaram valores significativamente menores para adiposidade (somatória das sete dobras cutâneas), peso corporal, IMC e força abdominal. Não foram encontradas diferenças significativas nas variáveis de velocidade e agilidade; quanto à força de membros superiores (MMSS), constatou-se que ela foi maior em quem não cumpriu a recomendação.

**Conclusões:**

Crianças que cumpriram a recomendação de atividade física apresentaram melhor composição corporal e força abdominal que as crianças que não cumpriram.

## INTRODUCTION

Physical fitness is the ability of human beings to perform physical daily work without harming their biological, psychological and social health, being a fundamental health indicator both at the individual and community level.^1^ Biological factors of physical fitness are anthropometric, metabolic and neuromotor variables.^2^ In broader terms, the higher the levels of physical activity (PA), the better the physical fitness of an individual.^3^ To the anthropometric extent, it would mean maintenance of body weight, decrease in body fat percentage and maintenance of body mass index (BMI), while to the metabolic extent, it expresses as higher values of oxygen consumption.^4^

Over the years, children and adolescents have spent less time PA and became sedentary. This contributes to the increase in body fat and to early onset of several chronic non-communicable diseases (NCDs).^5,6^ Evidence has shown that regular PA is inversely associated with overweight and obesity.^7^ Studies carried out over three decades with children showed an increase in adiposity and a linear increase in the mean of the seven skinfolds (SD) even when adjusted for nutritional status.^8^ In addition, not practicing enough PA was positively associated with worse eating habits and negatively associated with family income.^9,10^ In PA not being enough, screen time increases during free time, which leads to an impairment of health and motor performance.^11^

To cope with this situation, it is recommended that children and adolescents perform at least 60 minutes of moderate to vigorous physical activity (MVPA) daily, either continuously or cumulatively.^5^ When analyzing PA levels, children who do not take more than 9,500 steps per day show reduced health benefits.^10^

PA levels measurement has traditionally been performed by means of questionnaires,^12^ but this method can overestimate this variable. Recently, with the use of accelerometry, they can be measured more objectively and accurately.^13^ However, there are still very few national studies using the accelerometer for this purpose. Therefore, the objectives of this study were to describe and compare physical fitness variables according to compliance or non-compliance with PA recommendations, measured by accelerometry.

## METHOD

**Figure 1:**
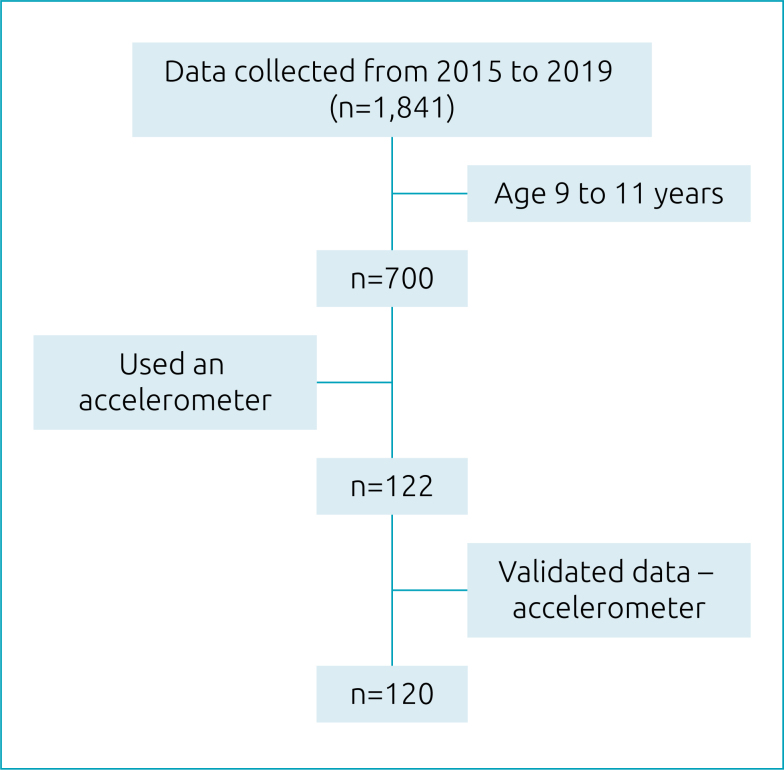
Flowchart of sample composition.

This study is a cross-section of the Mixed Longitudinal Project on Growth, Development and Physical Fitness of Ilhabela, developed by the Center for Studies of the Physical Fitness Research Laboratory of São Caetano do Sul (CELAFISCS), São Paulo, since 1978. All evaluations were carried out by healthcare professionals carefully trained in the measurement techniques used in the project. In total, 1,841 assessments were made between 2015 and 2019. After the inclusion criteria were applied—which involved a physical fitness assessment, being part of the public-school network, having used an accelerometer for at least five days and being apparently healthy—, 120 schoolchildren between 9 and 11 years of age were considered eligible (Figure 1). The project was approved by the Research Ethics Committee of Universidade Federal de São Paulo (Unifesp), under protocol n. 0056/10.

Among physical fitness variables, the anthropometric variables considered were weight (kg), obtained using a digital scale (Filizola^®^, Personal Life model), and height (cm), measured with a stadiometer with fixed base and mobile cursor. With these measurements, the BMI (kg/m^2^) was calculated.

Adiposity was measured with an adipometer (CESCORF, Analog Scientific Plicometer) based on the average of seven skinfolds (SFs) (mm): biceps, triceps, subscapular, suprailiac, mid-axillary, abdominal and calf. Waist and hip circumference (cm) were also measured, which allowed to calculate the waist-to-hip ratio (WHR).

Upper limb strength was established in kg using handgrip, dynamometry. A Takei dynamometer (Model Grip A T.K.K. 5001, Tokyo, Japan) was used. The subject was put in orthostatic position, holding the device in line with the forearm, parallel to the longitudinal axis of the body, and two measurements of each hand were taken alternately, considering the best execution of both, according to CELAFISCS^2^ standards. The Handgrip Strength (HGS) test through dynamometry is shown by studies to be an easy-to-apply form of measurement which can be used to monitor health status, in addition to showing a strong correlation with lower limb (LL) strength.^14,15^

LL strength was obtained by means of upper-limb assisted and non-assisted vertical jumping tests (AVJ) and by horizontal jumping (HJ, in cm). Agility was assessed with the shuttle-run test (sec). Speed was measured by the 50m (sec) sprint test. All measurements and tests followed the standards of CELAFISCS.^2^

The PA level was objectively monitored by an Actigraph GT3X-BT accelerometer. The device was worn on the waist in an elastic belt, in the mid-axillary line on the right side. The students were instructed to use the accelerometer 24 hours/day, for at least seven days, including two weekend days, and they only removed the device when bathing or when performing water activities.^16,17^ The minimum amount of data considered acceptable for analysis purposes was five days (including one weekend day), with at least 10 hours/day of use time. Sleep time was disregarded.

Data was verified using version 5.6 of the Actilife software (ActiGraph, Pensacola, FL, United States). Blocks of 20 consecutive minutes of 0 count were considered as non-use of device and discarded from the analyses. Data were collected at a sampling rate of 80Hz, downloaded in one-second periods and aggregated for 15-second periods.^16^ For data calculation, ≤25counts/15 seconds was used to define sedentary behavior (SB) and ≥574counts/15 seconds for MVPA,^17^ which provided the best classification accuracy between the cutoffs currently available for total SB and MVPA in schoolchildren.^18,19^

According to the results obtained for MVPA time, the sample was divided into two groups: “complies with recommendations”, made up of subjects meeting the MVPA^5^ recommendation of ≥60min/day, totaling at least 300 minutes per week; and “does not comply recommendations”, with <60min/day, less than 300min/week.

The Kolmogorov-Smirnov test was used to verify the normality of variables. The non-parametric variables had their data normalized by Blom's normal scores transformation. Mean, standard deviation (SD) and minimum and maximum values were used to characterize the sample according to sex in the descriptive analysis. To compare the means of groups, the Student's t test for independent samples was applied.

Comparison of physical fitness variables, according to compliance or non-compliance with PA recommendations, was verified by analysis of covariance (ANCOVA) adjusted for sex. The significance level was set at p<0.05 for all tests. Statistical analysis was performed using the software Statistical Package for the Social Sciences (SPSS) version 25.0.

## RESULTS

Table 1 shows that boys’ mean age was 10.1 years, and girls’ was 10.2 years. The groups had no significant differences in relation to weight, height and BMI.

**Table 1:** Sociodemographic description, physical activity level, sedentary behavior and physical fitness variables of schoolchildren according to sex.

	Boys					Girls					p-value
	n	Mean	SD	Min.	Max.	n	Mean	SD	Min.	Max.	
Age (years)^a^	57	10.14	0.74	9.03	11.89	63	10.18	0.68	9.13	11.82	0.073
Weight (kg)^a^	57	37.18	8.35	21.70	58.70	63	40.36	14.86	23.60	111.60	0.038
Height (cm)	57	139.60	8.40	120.00	158.00	63	142.85	10.61	117.00	164.00	0.064
BMI (kg/m^2^)^a^	57	18.97	3.42	14.15	26.44	63	19.38	5.06	11.22	45.86	0.077
Biceps SF (mm)^a^	57	8.48	4.18	3.00	18.80	63	9.16	4.07	3.60	20.00	0.012
Triceps SF (mm)^a^	57	13.76*	6.46	4.60	34.40	63	15.78	6.07	6.20	34.40	0.003
Subscapular SF (mm)^a^	57	10.34*	6.76	4.00	30.06	63	11.93	6.98	3.60	31.20	0.007
Suprailiac SF (mm)^a^	57	11.84*	9.21	3.10	39.50	63	13.82	9.01	4.20	42.60	0.003
Mean axillary SF (mm)^a^	57	9.35*	6.90	3.00	34.00	63	10.93	7.91	3.60	46.00	0.004
Abdominal SF (mm)^a^	57	16.81*	11.56	4.20	52.00	63	20.76	11.00	4.60	52.00	0.001
Calf SF (mm)^a^	56	13.22*	6.42	4.00	33.00	62	15.07	6.37	4.20	33.00	0.005
Average of the seven SFs (mm)^a^	56	12.03*	6.96	4.50	34.78	62	13.88	6.78	5.20	34.78	0.002
Arm circumference (cm)^a^	56	23.39	3.49	18.50	33.00	62	23.79	4.16	18.50	43.00	0.056
Relaxed arm circumference (cm)^a^	56	21.97	3.42	17.00	31.00	61	22.64	3.94	17.50	39.00	0.031
Waist circumference (cm)^a^	55	63.84	8.35	51.50	90.00	62	65.61	11.64	51.60	122.10	0.057
Hip circumference (cm)^a^	56	75.66	10.93	48.00	112.00	61	80.58	13.75	62.00	139.00	0.006
WHR^a^	55	0.85*	0.07	0.75	1.28	61	0.82	0.05	0.61	0.95	0.001
Calf circumference (cm)^a^	55	28.79	2.86	22.50	36.50	60	29.84	4.74	23.30	52.00	0.034
UVJ (cm)	57	25.24	6.28	8.00	39.00	63	24.73	5.40	11.00	38.00	0.064
AVJ (cm)^a^	57	25.19	5.41	12.00	36.00	63	23.94	6.25	6.00	38.00	0.022
HJ (cm)	55	147.56*	25.97	93.00	217.00	62	137.82	25.53	79.00	196.00	0.004
UL strength (cm)^a^	57	17.04	4.39	9.00	28.00	62	17.17	4.71	9.00	29.50	0.086
Abdominal strength (rep)	40	27.10	8.53	3.00	41.00	46	24.76	7.62	7.00	41.00	0.018
Flexibility (cm)	55	24.41	5.95	12.00	38.00	63	27.92*	6.07	12.00	42.00	0.001
Agility (seg)^a^	56	13.35	1.67	9.88	18.84	63	13.96	1.52	11.63	18.32	0.002
Speed (seg)^a^	55	10.60	1.35	8.49	14.44	61	11.03	1.64	8.75	17.54	0.009

a p<0,05; BMI: body mass index; SF: skinfold; WHR: waist-to-hip ratio; UVJ: unassisted vertical jumping; AVJ: assisted vertical jumping; HJ: horizontal jumping; UL: upper limbs.

Peripheral adiposity (mm) was higher in girls, verified by triceps, suprailiac, mid-axillary, abdominal and calf SFs (p<0.05). The other skinfolds had no statistically significant differences. Central adiposity (cm), verified by WHR, was significantly lower in girls (p<0.05) than in boys.

Boys’ horizontal jumping impulse was statistically superior to that of girls (p<0.05). However, upper limb and abdominal strengths did nor differ between sexes. As to other neuromotor variables, only flexibility and agility had statistically significant differences (p<0.05): girls were more flexible and boys were more agile.

The comparison of compliance with PA recommendations adjusted for sex is shown in Table 2. Those who met PA recommendations presented lower values of weight, BMI and, mainly, adiposity by the average of seven SFs (-38.1%), in relation to those who did not.

**Table 2:** Comparison of mean values of body composition and physical fitness of schoolchildren, according to compliance with physical activity recommendations (minimum of 300 minutes of moderate to vigorous physical activity/week).

		Complies			Does not comply			Δ%	p-value
		n	Mean	SD	n	Mean	SD		
Age (years)^a^		77	10.11	0.74	43	10.23	0.65	-1.19	0.041
Weight (kg)^a^		77	36.23	10.29	43	43.54	14.08	-20.18	0.001
Height (cm)		77	139.94	9.99	43	143.76	8.82	-2.73	0.070
BMI (kg/m^2^)^a^		77	18.22	3.31	43	20.90	5.39	-14.71	0.002
Biceps SF (mm)^a^		77	8.08	3.89	43	10.19	4.21	-26.11	0.002
Triceps SF (mm)^a^		77	13.65	6.04	43	16.92	6.32	-23.96	0.001
Subscapular SF (mm)^a^		77	9.59	5.76	43	14.02	7.87	-46.19	0.002
Suprailiac SF (mm)^a^		77	10.98	8.39	43	16.25	9.51	-48.00	0.001
Mean axillary SF (mm)^a^		77	8.42	5.57	43	13.33	9.25	-58.31	0.001
Abdominal SF (mm)^a^		77	16.46	10.23	43	23.22	12.19	-41.07	0.001
Calf SF (mm)^a^		75	12.91	5.79	43	16.43	6.94	-27.27	0.001
Average of the seven SFs (mm)^a^		75	11.42	6.04	43	15.77	7.49	-38.09	0.001
Arm circumference (cm)^a^		76	22.78	3.24	42	25.08	4.36	-10.10	0.001
Relaxed arm circumference (cm)^a^		76	21.54	3.34	41	23.76	3.94	-10.31	0.001
Waist circumference (cm)^a^		75	62.54	8.01	42	68.79	12.40	-9.99	0.001
Hip circumference (cm)^a^		76	75.49	10.75	41	83.30	14.43	-10.35	0.001
WHR^a^		75	0.83	0.05	41	0.83	0.09	0	0.098
Calf circumference (cm)^a^		75	28.44	3.15	40	31.02	4.79	-9.07	0.001
LL strength (cm)									
	UVJ (cm)	77	25.40	5.55	43	24.21	6.25	4.69	0.032
	AVJ (cm)^a^	77	24.74	5.52	43	24.17	6.51	2.30	0.085
	HJ (cm)	74	143.64	24.57	43	140.28	28.68	2.34	0.076
UL strength (cm)^a^		77	16.39	4.42	42	18.42	4.52	-12.39	0.002
Abdominal strength (rep)		51	27.43	7.63	35	23.54	8.30	14.18	0.005
Flexibility (cm)		75	26.57	6.11	43	25.79	6.51	2.94	0.021
Agility (seg)^a^		76	13.50	1.53	43	13.97	1.73	-3.48	0.029
Speed (seg)^a^		73	10.73	1.47	43	11.01	1.61	-2.61	0.047

BMI: body mass index; SF: skinfold; WHR: waist-to-hip ratio; LL: lower limbs; UVJ: unassisted vertical jumping; AVJ: assisted vertical jumping; HJ: horizontal jumping; UL: upper limbs. ANCOVA adjusted for sex;

a normalized variables.

Schoolchildren who complied with PA recommendations still obtained better results (p<0.05) in abdominal strength. However, paradoxically, HGS was significantly higher (p<0.05) in the group that did not comply with recommendations compared to those who did.

**Figure 2:**
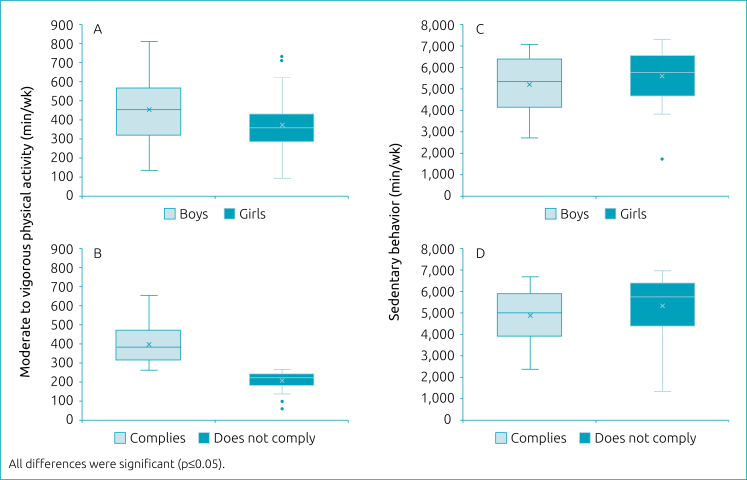
Time of moderate to vigorous physical activity (min/week) and sedentary behavior (min/week) according to sex and compliance/non-compliance with recommendations.

As Figure 2A shows, boys had higher mean values of MVPA (446.05±192.82min/week) than girls (351.02±144.19min/week). However, when performing an analysis per day of MVPA, boys did, on average, 89.21min/day, and girls 70.2min/day, which resulted in a difference of approximately +21.3% of MVPA for boys. However, as one can see in Figure 2B, when comparing subjects who comply with MVPA recommendations with those who do not, the mean total values of MVPA were the following: 486.05±146.52 *versus* 229.34±58.51min/week.

As for the sedentary behavior shown in Figure 2C, girls have statistically higher values (p<0.05) than boys (5,430.77±1,394.28 *versus* 4,907.10±1,557.80min/week). According to Figure 2D, those who comply with MVPA recommendations spent less time in sedentary behavior (4,907.10±1,557.80 *versus* 5,430.77±1,394.28), with a difference of −11.3%.

## DISCUSSION

Physical fitness is one of the most important markers for healthy growth and development in children, being influenced by PA levels. When measured objectively, verifying the intensity of PA is more accurate, resulting in more reliable data.^19^ In addition, this phenomenon can be observed from the comparison of the average values of body composition and physical fitness of schoolchildren considering compliance with PA level recommendations.^6^ This study described and compared the physical fitness variables according to compliance or not with PA recommendations, objectively measured by means of accelerometry.

As for the average MVPA, both boys and girls met PA recommendations, but boys accumulated more weekly and daily time, that is, they practiced 89.2min/day and girls practiced 70.2min/day, which corroborates data from the literature, in which it is established that boys are more active and accumulate more physical activities of greater intensity than girls.^17^ In a Finnish study, PA was also checked, but with lower values than in the present study, in which boys performed 58.2min/day and girls, 47.3min/day.^16^ However, these data are reversed on weekends, when boys usually do less PA than girls when measured objectively.^20^

Compliance with PA recommendations promotes several benefits, while non-compliance brings harm to health such as accumulation of peripheral and central fat, increased overweight, obesity and loss of cardiorespiratory fitness.^8,21^ Active children have 2% chance of becoming an active adult, while sedentary children have a 25% chance of becoming a sedentary adult.^22^

In our study, boys spent, on average, 981.42min/day in sedentary behavior, while girls spent 1,086.15min/day (p<0.04). This was also reported in another Finnish study^16^ with a sample aged 9 to 15 years, which assessed sedentary behavior by accelerometry. Boys spent, on average, 485.5min/day in this state, and girls, 511.0min/day (p<0.001).^16^ However, there are values higher than SB in relation to sex, with girls being more sedentary than boys.

When the present findings were adjusted for sex, schoolchildren who met the PA recommendations had −38.1% of adiposity, which corroborates the findings of a study that reported more active children with better indicators of body composition.^20^ This was also seen in earlier ages in a study with children aged 4 to 5 years that measured body composition by plethysmography.^20^ Data related to the same population of our study, from the Mixed-Longitudinal Project for Growth, Development and Physical Fitness of Ilhabela, showed that performing MVPA has a positive impact on adiposity, regardless of SB.^23,24^ Those who complied with PA recommendations showed a reduction in SB time by 11.3% compared to those who did not. Furthermore, the neuromotor variable had better results, demonstrated by abdominal strength 14.2% higher when comparing with those who did not comply with recommendations. A study carried out with Danish children reported that those who practiced sports had better aerobic conditioning and more lower limb strength when compared to children who did not practice any sports.^3^ The promotion of high-intensity PA for younger children may have beneficial effects on body composition and physical fitness, especially when it comes to muscle strength, in the long run.^23^ Previous research has already pointed that practicing sports also influenced better healthy lifestyle habits of participants.^25^

Compliance with PA recommendations did not lead to better results for upper limb muscle strength. This could be explained by the fact that muscle strength is a variable of late maturation.^26^ Sexual maturation is directly related to the increase in muscle strength, which usually occurs in the pubertal and post-pubertal phases,^27^ and this would be a plausible explanation for the differences in muscle strength observed in this study with children at earlier ages.^28^ It would be again important to emphasize that the influence of sexual maturation on muscle strength occurs in older age groups than in those assessed in the present study.

Some strengths of our study should be considered, namely the use of an objective measurement to measure PA levels through accelerometry. The period of use of the sensor to measure PA was approximately ten days for 24h/day, which allowed better accuracy of PA and SB variables. However, the study also had some limitations. Because of its cross-sectional design, it did not allow the establishment of a cause-effect relationship. The statistical analysis used for comparisons did not allow adjustments for some intervening variables such as nutrition, socioeconomic level and educational level of parents or guardians.

It can be concluded that MVPA recommendations are complied with by both sexes, but boys demand more time than girls. Children who complied with PA recommendations had better body composition and more abdominal strength compared to those who did not. However, children who did not comply showed greater upper limb strength.
